# mTOR favors senescence over quiescence in p53-arrested cells

**DOI:** 10.18632/aging.100164

**Published:** 2010-06-26

**Authors:** Thaddeus T. Schug

**Affiliations:** Laboratory of Signal Transduction, National Institute of Environmental Health Sciences, National Institutes of Health, Research Triangle Park, NC 27709, USA

**Keywords:** 06/22/10; accepted: 06/25/10; published on line: 06/26/10

**Figure 1.**
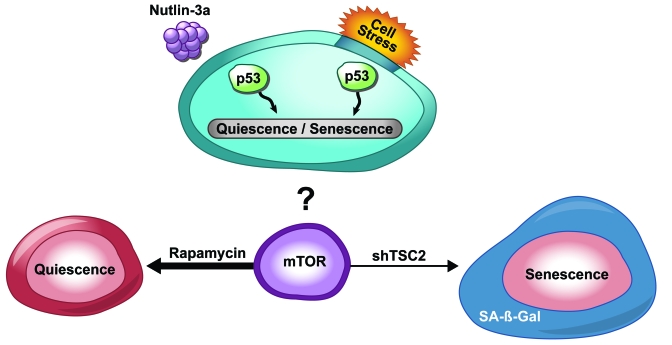
p53-induced senescence or quiescence. Cell stress factors or nutlin-3a activates p53. Rapamycin treatment inhibits mTOR signaling and cells enter a reversible quiescent state. ShTSC2-mediated activation of mTOR sends cells into senescence.

The p53 tumor suppressor protein controls cell fate by inducing apoptosis or cell cycle arrest in response to cell stress [1]. Cell cycle arrest can be temporary (quiescent) or permanent (senescent) depending on p53 regulation [2, 3]. Under normal conditions, p53 binds to MDM2, which transports the protein to the cytoplasm where it undergoes rapid proteosomal degradation. A class of chemotherapeutic molecules called Nutlins inhibit p53-MDM2 interaction, and can therefore be used to control p53 activity in cancer cells [4]. In this issue of Aging, Korotchkina et al make use of nutlin-3a to dissect the mechanism by which p53 induces cellular senescence and quiescence [5]. The group demonstrates that p53-mediated senescence is irreversible in cells that maintain mTOR (mammalian target of rapamycn) signaling. However, when mTOR signaling is inhibited, activation of p53 leads to quiescence (Figure 1). These findings may have broad implications because the mTOR pathway is dysregulated in many forms of cancer [6].

Establishing the mechanisms involved in cell cycle arrest and cell dormancy is critical for understanding cancer cell proliferation. Demidenko et al. have previously demonstrated that despite its ability to induce cell cycle arrest, in some cell types p53 is a suppressor, not an inducer of cellular senescence [7]. They have also shown that cells (HT-p21-9) induced into senescence using an ITPG-inducible p21 expression construct, were converted to quiescence in the presence of p53. In the same cells, nutlin-3a-induction of p53 caused reversible cell cycle arrest, and cells resumed proliferation after removal of nutlin-3a.

The same group has also demonstrated that when the cell cycle is blocked, activation of mTOR is required for induction of senescence. Addition of the TOR inhibitor rapamycin converted p21-induced senescent cells back to quiescence [7].  These findings suggest that activa-tion of p53 sets in motion cell cycle arrest, after which its ability to exercise senescence is dependent on its interaction with the mTOR pathway. Senescence is achieved if p53 is incapable of disabling mTOR. Therefore, activating both mTOR and p53 in order to achieve a permanent state of cell dormancy, may prove to be a promising therapeutic strategy for treating cancer. In their current study, Korotchkina et al further explore the role of mTOR as a senescence-inducing factor. They show that nutlin-3a-induced senescent cells converted to a quiescent state when mTOR was inactivated with rapamycin (Figure 1). Furthermore, the authors show that in p53-mediated quiescent cells, depletion of TSC2, a negative regulator of mTOR, results in conversion to senescence. This body of work may also offer explanations as to the role that p53 plays in aging. Others have shown that p53 function declines with age [8, 9], and mild activation of p53 may increase the lifespan of mice [10]. It will be interesting to further determine the interactions between p53 and mTOR in both models of cancer and aging.
